# Self-learning virtual organisms in a physics simulator: on the optimal resolution of their visual system, the architecture of the nervous system and the computational complexity of the problem

**DOI:** 10.18699/vjgb-25-110

**Published:** 2025-12

**Authors:** M.S. Zenin, A.P. Devyaterikov, A.Yu. Palyanov

**Affiliations:** Novosibirsk State University, Novosibirsk, Russia; A.P. Ershov Institute of Informatics Systems of the Siberian Branch of the Russian Academy of Sciences, Novosibirsk, Russia; Novosibirsk State University, Novosibirsk, Russia A.P. Ershov Institute of Informatics Systems of the Siberian Branch of the Russian Academy of Sciences, Novosibirsk, Russia

**Keywords:** virtual organism, computational modeling, computational complexity, vision system, neural network, simulator, PPO, reinforcement learning, Unity ML-Agents, виртуальный организм, компьютерное моделирование, вычислительная сложность, зрительная система, нейронная сеть, симулятор, PPO, обучение с подкреплением, Unity ML-Agents

## Abstract

Vision plays a key role in the lives of various organisms, enabling spatial orientation, foraging, predator avoidance and social interaction. In species with relatively simple visual systems, such as insects, effective behavioral strategies are achieved through high neural specialization, adaptation to specific environmental conditions, and the use of additional sensory systems such as olfaction or hearing. Animals with more complex vision and nervous systems, such as mammals, have greater cognitive abilities and flexibility, but this comes with increased demands on the brain’s energy costs and computational resources. Modeling the features of such systems in a virtual environment could allow researchers to explore the fundamental principles of sensorimotor integration and the limits of cognitive complexity, as well as test hypotheses about the interaction between perception, memory and decision-making mechanisms. In this work, we implement and investigate a model of virtual organisms with a visual system operating in a three-dimensional physical environment using the Unity ML-Agents software – one of the most high-performance simulation platforms currently available. We propose a hierarchical control architecture that separates locomotion and navigation tasks between two modules: (1) visual perception and decision-making, and (2) coordinated control of limb movement for locomotion in the physical environment. A series of numerical experiments was conducted to examine the influence of visual system parameters (e. g, resolution of the “first-person” view), environmental configuration and agent architectural features on the efficiency and outcomes of reinforcement learning (using the PPO algorithm). The results demonstrate the existence of an optimal range of resolutions that provide a trade-off between computational complexity and success in accomplishing the task, while excessive dimensionality of sensory inputs or action space leads to slower learning. We performed system performance profiling and identified key bottlenecks in large-scale simulations. The discussion considers biological parallels, highlighting cases of high behavioral efficiency in insects with relatively low-resolution visual systems, and the potential of neuroevolutionary approaches for adapting agent architectures. The proposed approach and the results obtained are of potential interest to researchers working on biologically inspired artificial agents, evolutionary modeling, and the study of cognitive processes in artificial systems.

## Abstract (ru)

Зрение играет ключевую роль в жизни множества различных организмов, обеспечивая ориентацию в пространстве, поиск пищи, избегание хищников и социальное взаимодействие. У видов с относительно простой зрительной системой, таких как насекомые, эффективная поведенческая стратегия достигается за счет высокой специализации нейронов, адаптации к конкретным условиям среды, а также благодаря дополнительным сенсорным системам – обонянию или слуху. У животных с более сложным зрением и нервной системой, таких как млекопитающие, когнитивные возможности и способности к адаптации значительно выше, однако выше и энергозатраты на работу мозга. Моделирование особенностей таких систем в виртуальной среде позволило бы исследовать фундаментальные принципы функционирования и обучения когнитивных систем, включая механизмы восприятия, памяти, принятия решений и их взаимодействие. В данной работе объектом исследования являются виртуальные организмы, обладающие зрительной системой и функционирующие в трехмерной физической среде на базе Unity ML-Agents – одного из наиболее высокопроизводительных современных симуляторов. Предложенная иерархическая архитектура управления, разделяющая когнитивные задачи между двумя модулями – зрительного восприятия/принятия решений и управления координированным движением конечностей для перемещения в физической среде – показала существенно большую скорость и эффективность обучения по сравнению с единой системой. Проведена серия численных экспериментов, направленных на выявление влияния параметров зрительной системы, конфигурации среды и архитектурных особенностей агентов на успешность их обучения с подкреплением (алгоритм PPO). Показано, что существует диапазон разрешений, обеспечивающий компромисс между вычислительной сложностью и успешностью выполнения задачи, а избыточная размерность сенсорных входных данных или пространства действий приводит к замедлению обучения. Должное внимание уделено также оценке вычислительной сложности системы и профилированию производительности ее основных компонентов. Полученные результаты представляют потенциальный интерес в контексте исследований искусственных агентов, вдохновленных биологическими системами, эволюционного моделирования, включая нейроэволюционные подходы для создания более адаптивных и умных агентов, и изучения когнитивных процессов в них.

## Introduction

Modeling cognitive activity, behavior, and evolutionary mechanisms in virtual environments constitutes an important step toward the development of artificial intelligence systems capable of learning, adaptation, and interaction with complex environments (Bongard, 2013; Stanley et al., 2019). The advancement of such systems has been facilitated by modern agent-based learning platforms, in particular Unity ML-Agents (Juliani et al., 2018), which allow the creation of fully featured three-dimensional simulations incorporating physics, vision, and multiple trainable agents.

Despite the relatively small number of neurons due to their small body size (compared, for instance, to mammals), the nervous systems of many invertebrates, including insects, exhibit remarkably complex, diverse, and adaptive behavior. For example, ants possess approximately 250,000 neurons, which is several orders of magnitude less than mammals (a mouse has about 7.1∙107), but these insects are capable of solving complex tasks of navigation, social interaction, coordination of collective actions, and route memory (Chittka, Niven, 2009). Moreover, according to a number of studies, certain species of ants are capable of passing the mirror test, a behavioral indicator of self-awareness (Cammaerts M.- C.T., Cammaerts R., 2015). This makes them unique among insects and highlights the potential of minimal but efficiently organized nervous systems, which are of considerable interest to modern science

Insect visual systems also serve as a source of inspiration for the design of artificial agents. In particular, compound eyes provide a wide field of view and high refresh rates, enabling efficient responses to rapidly changing stimuli (Land, Nilsson, 2012). However, their angular resolution is significantly inferior to that of humans, but this limitation is compensated by high sensitivity to movement and the capacity for learning at the level of entire behavioral sequences.

These considerations give rise to several fundamental research questions: what are the minimal requirements for an agent’s visual system that enable successful adaptation to its environment? What control architecture ensures cognitive modularity under constrained computational resources? In other words, how to construct an “artificial organism” – an agent with simple but functional elements of perception and decision-making. The present study addresses these questions by investigating virtual organisms endowed with vision and operating in a 3D environment, with a focus on their ultimate cognitive efficiency, scalability, and capacity for learning in tasks of search and navigation.

The interest in structures that enable movement with minimal design complexity is also evident in engineering systems. For example, a recent study (Song et al., 2022) examines the control of hybrid soft limbs, reflecting the pursuit of structurally simple but functionally efficient solutions for motion control. The body model of the virtual organism used in the present study, in terms of degrees of freedom and segment composition, is comparable to those employed in such constructions. This makes it possible to regard it as comparable in complexity to its physical counterparts

In our previously published work (Devyaterikov, Palyanov, 2022), we presented a simulator of the evolution of virtual organisms in a 3D environment, where each agent was equipped with a visual system and a neural network for processing sensory input. The system was based on a combination of neuroevolution and agent–environment interaction, enabling agents to perform elementary cognitive tasks that required the use of vision (such as searching for “food” necessary for “survival”) and allowing the assessment of agent survivability within a population. The present work provides estimates of the computational complexity of calculations related to physics (agent bodies, the environment, and their interactions), first-person 3D rendering for each agent, and the operation of their neural networks. In addition, it introduces a new hierarchical agent model and presents the results of a quantitative analysis of training time, speed, and efficiency as a function of visual system resolution. The (Aksoy, Camlitepe, 2018) study provides data on the number of ommatidia (photosensitive sensors) for various ant species (from 100 to 3,000). Roughly approximating such vision with a square pixel matrix, this corresponds to a visual resolution from 10 × 10 to 55 × 55

The present work combines reinforcement learning methods (PPO (Schulman et al., 2017)), convolutional neural networks (O’Shea, Nash, 2015), approaches to hierarchical agent training (Vezhnevets et al., 2017), and practical analysis of resource-saving simulation schemes (Peng et al., 2018). We demonstrate that a hierarchical agent approach (e. g., a “Walker/Searcher” pair) enables more stable and interpretable behavior while reducing training time at a comparable level of task complexity

Particular attention is given to investigating the impact of visual system resolution on agent learning rate, with an assessment of the minimal input image size at which the ability to perform visual search and navigation tasks is preserved. Such investigations are relevant both for biologically inspired modeling and for the development of compact and efficient AI agent architectures capable of functioning under limited computational resources (Hassabis, Humaran, 2017; Zador, 2019).

In addition, this study examines the effect of task decomposition strategies (navigation and locomotion) on training efficiency. This approach provides deeper insights into the principles underlying cognitive modularity and distributed control in complex agent systems (Botvinick et al., 2020; Tschantz et al., 2020). The introduced Searcher agent, relying exclusively on visual perception, interacts with the Walker agent, responsible for physical movement. Such a scheme enhances the adaptability of the model and improves the interpretability of agent behavior.

Thus, the aim of the present work is to conduct a systematic investigation of the limits of cognitive complexity in agents equipped with visual systems, to develop optimal control architectures and perceptual parameters, and to evaluate the performance and scalability of the proposed system implemented on the Unity ML-Agents platform.

## Materials and methods

Problem statement. The problem under consideration is formulated in terms of a Markov decision process, where the agent interacts with a three-dimensional physical environment and learns to maximize cumulative reward. The task performed by the agent is described below:

Environment E: a square arena bounded by walls. Targets with radius r appear randomly within the arena and must be collected. Once a target is reached, a new one is generated

Agent state st: consists of an RGB image from the first-person camera of size h × w × 3, long with a vector of control parameters (joint angles of the limbs and the corresponding torques).

Agent action at: a single scalar value representing a normalized rotation angle in the interval [−1, 1]. This parameter determines the direction of the agent’s body movement. The actual rotation angle is defined as θ = at * θmax, where θmax is the maximum allowable rotation angle specified in the experimental parameters. In different experimental series, various values of this parameter were used, which allowed us to investigate its impact on policy efficiency (results are reported in Section “Results with varying rotation angles”). The restriction to a single control variable is due to the fact that low-level locomotion tasks (coordination of limbs and balance maintenance) are delegated to a separate Walker module, enabling the focus to remain on the cognitive aspects of the task, i. e., perception and decision-making

Reward function R(st, at): an agent receives a positive reward for successfully reaching the target.

Objective: to maximize the cumulative reward over an episode of time T, i. e., to develop a policy that enables efficient navigation in the environment and target collection based on visual information

One of the goals of our study is to identify the minimal input image resolution at which the agent can still successfully learn within a reasonable amount of time. The formal problem formulation is as follows:

Training success is defined as achieving an average reward of at least Rgoal = 5 per episode (where the reward is granted for target collection by the agent). The value of Rgoal was determined experimentally. As shown in the training results (see Section “Dependence of learnability on image resolution”), an untrained agent, due to random wandering, attains on average no more than 2.

Training time of the agent until reaching the threshold value: T(N ) ∈ ℝ+

Average reward R(Res, T ) achieved by the agent after training with input resolution Res = h × w × 3 over time T.

Admissible set of resolutions Res ∈ ℕ, from 20 × 20 × 3 to 100 × 100 × 3 with a step of 20 and with an additional case of 84 × 84 × 3, used as the default resolution in Unity MLAgents

It is required to find minr ∈ ResT(N ), where R(Res, T ) ≥ Rgoal, that is, the minimal training time over admissible resolution for which the achieved reward meets or exceed the threshold Rgoal.

Simulator architecture. The proposed system employs a hierarchical control architecture for the agent, separating perception and motion functions across two levels. The lowerlevel agent (Walker) is responsible for physical locomotion in the environment, relying on local sensors and a pre-trained locomotion model. The higher-level agent (Searcher) receives visual input from the camera and decides on the movement direction, transmitting a control signal to the Walker agent in the form of a normalized rotation angle. This approach makes it possible to isolate the complex problem of sensorimotor transformation (from image to action) from the tasks of motion stabilization and limb coordination. As a result, training of the Searcher becomes faster and more stable, since it controls only a single variable. The internal communication between agents is implemented within the Unity environment through the transmission of the direction parameter to the Walker controller. In the training mode, the Searcher agent processes visual data and generates a rotation angle, which is used as the control parameter for selecting the body orientation at the next step. The Walker, in turn, executes the specified direction, ensuring movement in the intended direction.

During simulation, the environment is dynamically updated: after a target (a unit of “food” required for survival) is collected by the Searcher agent, a new one is generated at a random position (to maintain the number of available “food” units at a constant level). When the agent falls or the maximum number of steps is reached, the episode is reset. The architecture supports parallel execution of multiple environments, each containing one Searcher and one Walker, which enables training to be scaled within the Unity ML-Agents framework

Simulation environment. For the experiments, we selected the modern Unity ML-Agents platform, which demonstrates high performance and provides convenient tools for building complex three-dimensional simulations with reinforcement learning integration. Unity also offers built-in support for parallel environments, visual sensors, and integration with the PyTorch library

Each environment represents a bounded square arena (DynamicPlatform) with walls, a floor, and randomly placed targets that the agent must collect. The platform size is fixed, and the target spawn coordinates are uniformly sampled across the available area. When the agent collides with a target, it disappears and is immediately replaced by a new one. The walls are impenetrable and serve as physical boundaries of the environment.

Simulation parameters are specified via the CrawlerSettings component and include the simulation tick rate of the physical world, gravity, episode duration (max_step – the number of simulation steps at which the agent receives observations and performs actions), and the number of parallel environments. If the agent falls (detected by body contact with the floor), the environment is automatically reset. Each parallel environment contains one Searcher agent, embedding a nested Walker, equipped with an individual camera mounted at the front of the head, which supplies the agent’s neural network with a stream of first-person visual information

**Fig. 1.**
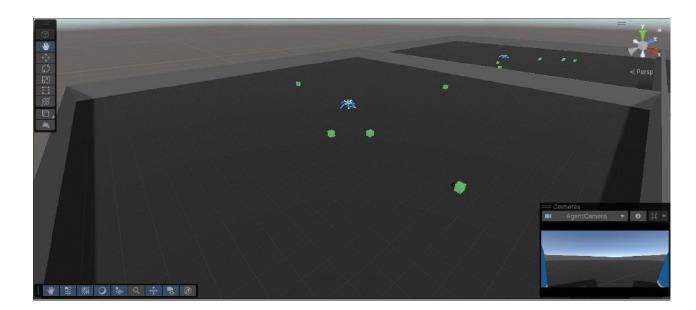
Unity model of the Walker agent, with the first-person camera view shown in the bottom right corner. Two environments, the agents, and a number of targets are also presented.

The number of simultaneously running environments (num_envs) depended on the agent type: for the Walker agent, which does not use visual input, 10 environments were employed, while for the Searcher agent, four environments were used. This configuration enabled efficient utilization of GPU resources and accelerated data collection through parallel interaction with the environment. For each environment, actions data, observations, and rewards were collected independently and synchronized with the training strategy in Python via the Unity ML-Agents gRPC interface. Figure 1 presents a view of the simulation from the observer’s perspective, showing two environments, the agents, and a number of targets.

**Fig. 2.**
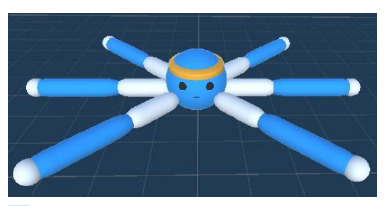
Walker agent model in Unity.

Walker agent model. The lower-level agent (Walker) is a complex articulated model with six limbs, implemented in the Unity engine using the Rigidbody and ConfigurableJoint components. Each limb consists of two segments: upper and lower – with three degrees of freedom (resulting in a total of 18 degrees of freedom for all legs). This design enables the agent to perform realistic locomotion and maintain stability during movement. The agent model in the Unity environment is shown in Figure 2.

**Fig. 3.**
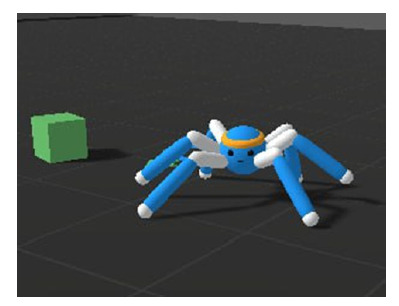
Walker agent model in motion.

The control system is implemented through the JointDrive Controller module, which converts control signals into desired joint angles and forces. The control parameters are represented as a vector of dimension 30: 18 values control joint angles, and 12 correspond to the torques applied to them. Specifically, for each of the six legs, the upper segment is controlled by two angles (rotation about the X and Y axes), and the lower segment by one angle (rotation about the X axis), yielding 18 control parameters in total. In addition, for each of these 12 segments, a control force is specified, determining the intensity of movement, which yields another 12 parameters. At each step, the agent receives observations that include information on current joint angles, velocities, surface contacts, target direction vector, body orientation, and ground raycast data. The Walker agent model in motion is shown in Figure 3.

**Fig. 4.**
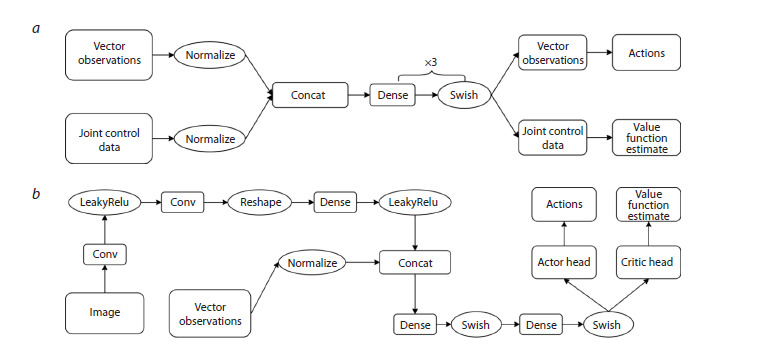
Schematic representation of the Walker (a) and Searcher (b) agent’s neural network architecture.

The neural network architecture of the Walker consists of three fully connected layers with LeakyReLU activation functions and two outputs: an actor (30 action parameters) and a critic estimating the value function (Fig. 4a). The input layer has a dimensionality of 223 (vector features and joint parameters), while the hidden layers each contain 512 neurons. The total size of the model is 655,903 parameters and 1,567 neurons, making it lightweight enough for real-time training.

The reward function for the Walker agent is defined based on the deviation of the agent’s current body velocity from the target velocity and the alignment of its movement direction with the specified vector. This enables the agent to learn purposeful locomotion in the desired direction while maintaining physical stability. After training, the Walker agent is used in inference mode as part of the Searcher agent, providing stable execution of movement

During training, the critic block receives the same input as the actor – the state feature vector. Based on these data, it learns to approximate the expected cumulative reward the agent will obtain in the future if it continues to act according to the current policy. At the early stages of training, this estimate is inaccurate, but it is gradually refined through backpropagation of the error, grounded in the actual rewards received by the agent. Thus, the critic does not initially “know” what is good or bad – it learns to distinguish this by comparing predicted rewards with the real rewards accumulated during simulations

After training, the Walker agent is used in inference mode as part of the Searcher agent, ensuring stable motion execution based on the deviation of the current body velocity from the target and the alignment of the movement direction with the specified vector. This allows the agent to learn purposeful locomotion in the desired direction while maintaining physical stability.

Searcher agent model. The higher-level agent (Searcher) is responsible for perceiving the environment and selecting the direction of body movement. Unlike the Walker agent, it does not interact directly with the physical components of the simulation but instead controls the Walker by transmitting a normalized rotation angle in the interval [−1, 1]. Thus, the Searcher serves as a cognitive module that implements a target-search strategy based on visual information. The primary input source for the Searcher agent is the image obtained from a camera mounted on the agent’s body (at the front of the head). The camera is oriented forward and positioned at a height corresponding to the head of the virtual organism. The image resolution varies across experiments from 20 × 20 to 100 × 100 pixels, with increments of 20 in each dimension (three-channel RGB), allowing for analysis of the impact of visual load and frame resolution on the model’s learning performance

For image processing, a convolutional neural network is employed, consisting of two convolutional layers (Conv2D), a flattening layer (Flatten), and subsequent fully connected layers. The output of the visual input processing is concatenated with vector observations and fed into two output layers: the actor (a single value representing the rotation angle) and the critic (value function estimate). The activation functions used are LeakyReLU and Swish. A schematic representation of the Searcher agent’s neural network architecture is presented in Figure 4b

The Searcher agent is trained using the Proximal Policy Optimization (PPO) algorithm with a continuous action space. The objective function is to maximize the cumulative reward for collecting targets in the arena. Upon colliding with a target, the agent receives a positive reward; upon colliding with a wall or remaining inactive, it is penalized. When max_step is exceeded or the body falls, the simulation episode terminates and a new one begins

Unlike the Walker agent, which is pre-trained once and then used only to execute the learned behavior (inference mode), the Searcher agent is trained from scratch, and its neural network includes image processing, which increases computational costs but enables the realization of biologically plausible behavior based solely on visual perception. This makes it possible to model cognitive constraints and analyze the impact of visual resolution on the speed and stability of learning.

Training algorithms and hyperparameters. The PPO algorithm is a gradient-based policy optimization method that belongs to the family of actor-critic approaches. Such methods combine the training of a policy and a value function. By avoiding abrupt policy updates, in contrast to classical methods of this type, PPO is designed to improve the stability and reliability of training. The Actor, the component responsible for selecting an action in each state, implements the agent’s policy. The Critic, in turn, evaluates how good the chosen action was by using the value function. This approach combines the advantages of stochastic action selection (important for exploration of the environment) with the evaluation of these actions based on accumulated experience

The PPO algorithm operates within the framework of a Markov decision process (S, A, P, R, γ), where S – the set of states, A – the set of actions, P(s′ | s, a) – the state transition probability, R(s, a) – the reward function, γ ∈ [0, 1] – the discount factor

The parameterized policy πθ(a | s) defines the probability of selecting action a in state s, where θ signifies the parameters of the actor neural network. The critic Vϕ(s) is an approximation of the value function V π(S) = E[Rt | st = s], with parameters ϕ, where Rt = rt + γrt+1 + γ2rt+2 + … is the discounted sum of future rewards. In PPO, instead of direct gradient updates, the so-called clipped objective function is used:

**Function. 1.**
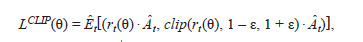
Function. 1

where: rt(θ) = πθ(at |st) πθOld (at |st) – the probability ratio between the new and the old policy, ε ∈ (0, 1) the clipping parameter, typically ε = 0.1 or 0.2,At – the advantage estimate.

If the new action deviates too strongly from the old one (i. e., rt falls outside the interval [1 – ε, 1 + ε]), the gradient is suppressed. This prevents abrupt changes in the policy.

To estimate At, the generalized advantage estimation (GAE) is used:

**Function. 2.**
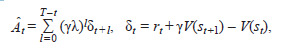
Function. 2

where λ ∈ [0, 1] – is the smoothing parameter. This method improves training stability by reducing variance.

The loss function in PPO consists of: • the policy loss LCLIP, • the value critic loss (MSE between the predicted V(st) and the target value), • an entropy bonus to encourage action diversity:

**Function. 3.**
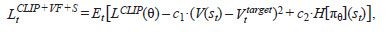
Function. 3

where H [π] is the policy entropy and c1, c2 are the corresponding coefficients.

A schematic representation of the proximal policy optimization algorithm is shown below

**Algorithm. 1.**
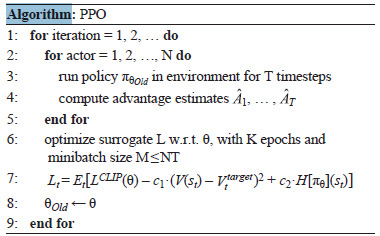
Algorithm. 1

where N is the number of parallel actors collecting data over T time steps, and K is the number of epochs. Neural networks are used to approximate the target policy and the value function.

The choice of PPO in this work is motivated by several factors: the algorithm supports continuous action spaces, which is critical for the locomotion of virtual organisms with multi-joint limbs. The update constraint allows the agent’s policy to evolve incrementally without disrupting previously learned behaviors. PPO can also be effectively applied in architectures incorporating convolutional neural networks (CNNs) that process images from the agents’ cameras. In addition, the Unity ML-Agents environment provides a builtin PPO implementation, which simplifies configuration and accelerates the cycle of computational experiments

The actor network receives state features (velocities, joint positions, surface contacts, etc.) together with visual data processed through convolutional layers. The agent’s objective is to maximize the reward associated with locomotion and stability while moving in the chosen direction. PPO enables smooth adaptation of the policy to complex dynamics and noisy feedback from the environment

For the Walker agent, the action space is represented by a vector of 30 continuous values (18 for joint angles and 12 for actuation forces/torques controlling joint movements), whereas the Searcher agent controls only a single parameter – the movement direction (a normalized rotation angle in the range [−1, 1]). Both models are trained asynchronously using multiple parallel environment simulations (from 4 to 10), which enables efficient data collection and accelerates the optimization process

The main training parameters are (detailed in the documentation (Juliani et al., 2018)):

• algorithm: PPO (proximal policy optimization); • framework: Unity ML-Agents + PyTorch backend; • learning_rate: 3 × 10–4. A coefficient that determines the step size when updating neural network parameters; • batch_size – the size of the data batch used for one training step: Searcher: 1,024, Walker: 2,048; • buffer_size: 10,240. The number of environment interactions used for one training cycle. Configured as a multiple of batch_size × num_envs; • num_epochs: 3. The number of optimizer passes (epochs) over one data buffer before it is updated; • gamma (discount factor): Searcher: 0.99, Walker: 0.995; • lambda (GAE): 0.95; • clip_range: 0.2.

The Walker agent was trained separately in an isolated environment until stable and straight locomotion was achieved. The average number of steps to convergence was approximately 2–3 million. After this stage, the model weights were fixed, and the agent was used only in inference mode.

The Searcher agent was trained independently of the Walker. The average number of steps per experiment ranged from 5 to 10 million, depending on the environment configuration (camera resolution, max_step, number of target objects in the environment, etc.).

Simulation parameters were specified through YAML configurations of ML-Agents. To ensure stable and reproducible results, a fixed parameter was used to set the initial value for the random number generator applied in both the environment and training (random_seed), along with consistent settings: when the number of environments (num_envs) was changed, buffer_size was necessarily adjusted proportionally, as required by the ML-Agents framework

All experiments were conducted on a computer equipped with a CUDA-compatible GPU (see Section “System performance and profiling”). The software versions used were: Unity 2022.3, ML-Agents 21.0, PyTorch 2.0.1, and Python 3.10

Experiments. The experimental part of the study (numerical experiments) was aimed at investigating the influence of visual system parameters, environment configuration, and architectural constraints on the training efficiency of agents. All experiments were carried out in isolated environments using a fixed Walker agent model and a trainable Searcher agent. The main directions of investigation were as follows:

1. Impact of camera image resolution on learnability. A range of resolutions was considered: 20 × 20, 40 × 40, 60 × 60, 80 × 80, 84 × 84 (the default resolution for Unity MLAgents), and 100 × 100 pixels. For each of these, a separate training of the Searcher was conducted under otherwise identical parameters. The objective was to determine the minimal resolution at which the agent consistently achieves the target behavior (Reward ≥5).

2. Impact of speed control capability. In one of the experiments, the Searcher agent was additionally given the ability to control the target movement speed (a second continuous output parameter). The objective was to determine whether this would lead to more flexible behavior or instead complicate the learning task.

3. Variation of maximum rotation angle. The Searcher agent transmits a body rotation command. In different experiments, the maximum allowable rotation angles were tested: 90, 120, 180, and 270°. The hypothesis examined was that larger angles may simplify navigation but make the behavior less precise and stable.

4. Impact of episode length (max_step parameter). In the experiments, two values of the max_step parameter were considered: 5,000 and 20,000. The value max_step = 5,000 was used as the baseline, as it allowed the agent to receive rewards quickly enough and provided timely feedback to the learning algorithm. The value 20,000 was considered as an alternative, applicable to tasks with longer action sequences and delayed rewards.

5. Verification with manual control. To validate the behavior of the trained Walker model, manual control of the agent was implemented (via the A/D keys, left/right). This made it possible, on the one hand, to confirm that the observed effects (e. g., halting of movement) were caused by body dynamics rather than the Searcher agent’s policy, and on the other hand, to test whether a human, using the same type of control, could successfully perform the target-search task (an assessment of controllability and environment perception).

All experiments were recorded using the Unity ML-Agents logging system and analyzed in TensorBoard, a visualization tool for monitoring the training process that allows real-time plotting of reward dynamics, loss functions, simulation speed, and other metrics. The success criteria are described in Section “Problem statement”.

## Results

**Dependence of learnability on image resolution**

**Fig. 5.**
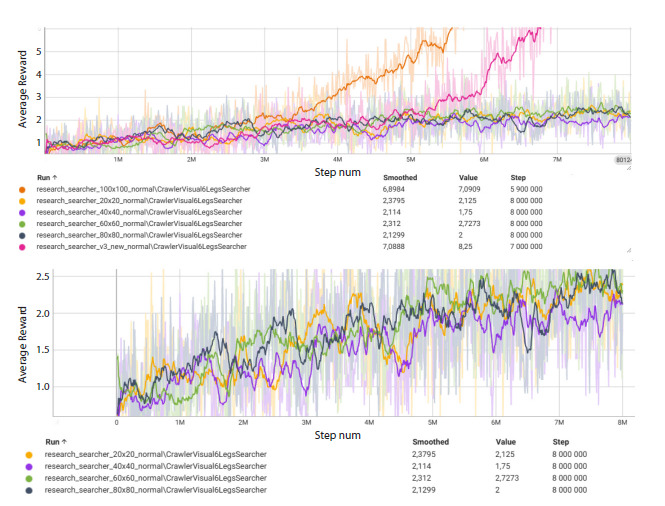
Training results of the Searcher agent at different input image resolutions The upper panel shows the average reward curves for all investigated resolutions; the magenta curve corresponds to 84 × 84, and the orange curve to 100 × 100. The lower panel presents the same data with the dominant curves removed, allowing a more detailed view of the remaining variants (20 × 20, 40 × 40 и 60 × 60, 80 × 80)

The results of the series of experiments with different input image resolutions showed that the minimal resolution at which the agent consistently achieved the target behavior (average reward ≥5) was 84 × 84 pixels. At resolutions of 20 × 20, 40 × 40, and 60 × 60, training required substantially more time, although the trend toward improvement was preserved. The resolution of 100 × 100 also allowed the target reward to be reached, but training at 84 × 84 was slightly faster due to lower computational load. The results of this experiment are presented as TensorBoard plots in Figure 5.

**Impact of speed control on training**

**Fig. 6.**
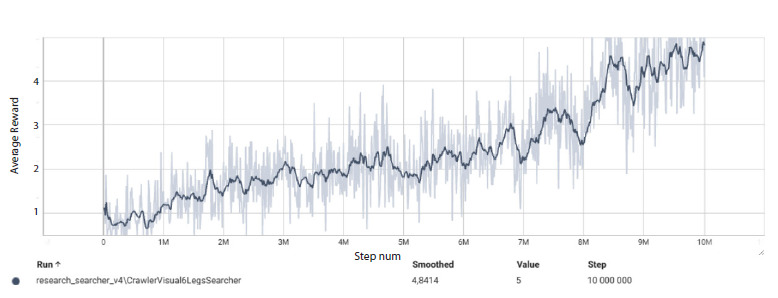
Training results of the Searcher agent with input image resolution 84 × 84 and the addition of a second control parameter (movement speed) alongside the primary one (rotation angle).

The addition of a second control parameter (movement speed) increased the dimensionality of the action space and significantly complicated training. The agent required more time to converge (approximately 33 % longer under otherwise identical conditions), and the resulting behavior was less stable – for the given task, speed control is largely a redundant parameter. This supports the simple hypothesis that increasing the number of degrees of freedom requires a more complex policy and hinders model training. The results of this experiment are shown as a TensorBoard plot in Figure 6.

**Results with varying rotation angles**

The best results were obtained with a maximum rotation angle of 90°. Increasing the angle to 120° led to a slight decrease in stability, while at 180 and 270°, the agent did not reach the target reward level, requiring longer and less efficient training. This indicates that an excessively wide action space hinders the development of a stable navigation policy.

**Impact of episode length (max_step)**

With max_step = 5,000, the agent demonstrated stable training, receiving timely feedback on goal achievement. Increasing the episode length to 20,000 did not improve training quality, while simulation time and resource load increased. Therefore, max_step = 5,000 was used as the primary setting, as it provided a balance between training efficiency and computational cost

**System performance and profiling**

To evaluate the scalability and computational efficiency of the simulator, profiling of key system components was conducted under varying visual sensor resolutions and numbers of parallel environments. All measurements were performed on a machine equipped with an NVIDIA GeForce RTX 3070 GPU and an AMD Ryzen 5 7500F CPU (6 cores, 12 threads, 3.7 GHz base clock, 5.0 GHz in turbo mode).

The contribution of main simulation components to computational costs:

• Physics Engine – less than 1 ms per step, virtually independent of resolution; • graphics and sensors (Camera.Render, PostProcess) – from 3.2 to 9.5 ms depending on resolution (almost linear dependence); • neural network (PyTorch Inference) – approximately 35 ms per step when using convolutional architecture for Searcher; • Unity–Python communication (gRPC, serialization) – from 45 to 60 ms. With an increasing number of agents, this component becomes one of the main system bottlenecks, since communication costs (serialization/deserialization, data exchange) grow proportionally to the number of agents; • other (UI, garbage collection, VSync) – up to 20 % of runtime, may increase during active debugging.

At a resolution of 84 × 84 with four parallel agents, the average simulation step time was approximately 3.6 ms, corresponding to about 278 steps per second. At a resolution of 100 × 100, the step time increased to 3.8 ms, reducing performance to roughly 263 steps per second. All measurements were conducted without scene visualization. In all experiments with the Searcher agent, the number of simultaneously running environments was set to 4.

Thus, the main limiting factor in scaling is not physics or rendering, but data exchange between Unity and Python. This should be considered when planning large-scale experiments or transitioning to population-level modeling. A working prototype for reproducing the results is available in the repository at: https://github.com/DerpyFox/organism_simulator.

## Discussion

**Results interpretation**

The obtained results demonstrate that the success of training agents with visual perception directly depends on the resolution of the input image. Too low a resolution (up to 60 × 60) leads to a loss of spatial structure of the scene and the agent’s inability to develop a stable strategy. On the other hand, resolutions above 84 × 84 do not provide a noticeable gain in efficiency but increase the computational load. This confirms the existence of an optimal range of visual perception, comparable to that evolutionarily formed in insects: their vision developed to be sufficiently detailed for performing behavioral tasks (Chittka, Niven, 2009).

Despite the observed dependence between visual system resolution and the success of agent training, it should be noted that in nature there are organisms capable of effective behavior even with extremely low visual resolution. For example, in some ant species, as mentioned in the introduction, the visual system is comparable in scale to a resolution of about 10 × 10, yet this does not prevent them from confidently navigating, locating food, interacting with their environment, and even passing the mirror test (Cammaerts M.-C.T., Cammaerts R., 2015). Such efficiency is determined not only by vision but also by the developed olfactory system, which plays a key role in perceiving the surrounding world. In addition, the neural systems of real insects may possess a range of properties that enhance their effectiveness. These systems were shaped through long evolutionary processes and are adapted to specific living conditions and the typical tasks of a living organism – for example, navigating in complex environments, searching for food, and interacting with conspecifics. They exhibit a high degree of neuronal specialization and mechanisms of adaptation to changing stimuli. Such “tuning” to real-world conditions makes it possible to efficiently process even limited or fragmentary sensory signals, including visual, olfactory, and mechanosensory inputs.

The addition of speed control and the increase in rotation angle showed that even a slight expansion of the action space leads to slower learning. Thus, it is important to maintain a balance between the expressiveness of the model and its learnability. The division of perception and body control tasks between the Searcher and Walker agents proved to be critical for achieving stable behavior.

**Biological parallels and cognitive efficiency**

The results resonate with principles observed in insects: minimal but functionally redundant visual systems enable successful navigation and real-time decision-making. Similarly, the proposed architecture allows the agent to achieve target strategies with limited resolution and a relatively small neural network.

When the obtained results are considered in the context of real biological systems, a parallel can be drawn with the evolutionary trade-offs that arise between sensory accuracy, computational cost, and behavioral adaptability. For example, the visual systems of insects such as fruit flies (~150,000 neurons) or honeybees (~960,000 neurons) provide basic object recognition and spatial orientation with a minimal number of neurons and extremely limited bandwidth (Menzel, 2012). These organisms do not possess high-resolution visual systems, but they achieve high efficiency through a combination of rapid response, sensorimotor architecture, and decisionmaking strategies (Chittka, Niven, 2009). Such considerations are well illustrated by insects with a high level of social organization. In ants, division of labor and communication are shaped not only as innate behavioral patterns but also as the result of flexible adaptation at the level of individual workers. The distribution of roles within a colony may vary depending on age, physiological state, and the current situation, while information transfer between ants is achieved through a wide range of signals (Chittka, Muller, 2009). Thus, even simple agents with limited cognitive capacities can achieve high efficiency through the organization of interactions and simple behavioral rules

Agents in our simulator demonstrate the ability for adaptive behavior even at relatively low visual resolutions (e. g., 84 × 84 pixels), which allows further analogies to be drawn with minimal cognitive systems in nature. Such models can be employed as artificial systems that reproduce key behavioral aspects of simple organisms and serve as a basis for generating hypotheses about the neurophysiological mechanisms of perception and behavior in invertebrates.

**System limitations**

The main limitation of the system lies in the communication overhead between the Unity environment and the PyTorch training framework. Even with high computational performance of the processing units, serialization and data transfer via gRPC become the bottleneck. In addition, at this stage, the environment remains limited in complexity: it lacks obstacles, dynamic topography, and inter-agent interactions. Finally, the agent architectures are fixed and do not undergo evolution or temporal adaptation (only parameter weights change, while network topology remains unchanged).

**Future directions**

Further development is possible in several directions. The introduction of neuroevolutionary mechanisms (e. g., the NEAT approach – NeuroEvolution of Augmenting Topologies) would make it possible to investigate not only changes in neural network weights but also the evolutionary optimization of network structure. This is particularly relevant in the context of energy costs: with excessive brain complexity, resource consumption increases, whereas in simpler environments it may be advantageous to reduce the number of neurons. In this way, agents could autonomously adapt the size and potentially the architecture of their neural networks, reducing redundancy under conditions of low cognitive load. In biological systems, even a slight increase in nervous system complexity can lead to a noticeable rise in energy expenditure. For example, in the fly Calliphora vicina, the retina alone consumes about 8 % of the organism’s resting metabolic rate (Niven, Laughlin, 2008). In humans, by contrast, the brain accounts for only about 2 % of body mass yet consumes up to 20 % of the body’s energy (Attwell, Laughlin, 2001). These data indicate that the benefit of reducing the number of neurons or decreasing the complexity of the sensory system can be substantial.

Introducing environmental elements involving resource competition (multiple agents, a limited number of targets, and the ability of more advanced agents to select and solve more complex cognitive tasks from those available in the system, thereby gaining additional advantages) would make it possible to analyze behavioral strategies at the population level.

A promising direction is the addition of an olfactory model – a sensory channel based on short-term “traces” in the environment, analogous to pheromone markings in ants. Such traces may decay over time, differ in content (e. g., distinguishing between a satiated and a hungry ant), and influence an agent’s trajectories, thereby reinforcing elements of indirect communication and collective behavior. It would also be reasonable to incorporate memory and recurrent modules into the Searcher model to study navigation under partial observability

## Conclusion

This study was aimed at the quantitative and qualitative evaluation of architectural and sensory parameters in the task of training visually guided agents in a three-dimensional simulation. We proposed and implemented a hierarchical control model in which the locomotion agent (Walker) functions as a low-level executor of movements, while the perception and navigation agent (Searcher) makes strategic decisions based on visual information.

A systematic analysis demonstrated that even under limited sensory input (due to low resolution), agents are capable of developing stable behavioral strategies, provided that the model and environmental conditions are designed with cognitive load in mind. It was established that a resolution of 84 × 84 pixels offers a compromise between computational efficiency and minimal cognitive adequacy, whereas increasing the dimensionality of the action space without a corresponding increase in training resources leads to degraded performance.

Our results support the hypothesis that minimally complex neural network agents can realize sophisticated behavioral patterns under conditions of limited sensory perception, where the agent receives only partial information about the environment. These findings are consistent with observed examples of cognitive efficiency in invertebrates, such as ants and bees, and open up prospects for the use of such models in biological modeling, robotics, and research in the field of neuroevolution

In the future, the system may be extended toward population- level simulations incorporating competition, interagent communication, and strategy adaptation in a changing environment. The architecture can be further enhanced with memory modules, recurrent connections, or neuroevolutionary mechanisms, enabling the study of more complex cognitive phenomena in virtual populations.

It was also shown that the use of visual information, despite its expressiveness, requires substantial computational resources and, in some cases, may be less efficient than simpler sensory models. These observations highlight the importance of sensory architecture choice when designing minimally sufficient cognitive agents

Another key finding was the recognition of the critical role of environment design and training structure in the success of modeling. Initial attempts to train behavior through a single neural network model that combined locomotion and strategy did not lead to the emergence of the ability to detect and collect targets (“food” units), due to difficulties in balancing rewards and formulating the task. The introduction of a functionally separated approach (search and locomotion) made it possible to achieve a substantial improvement in learnability and behavioral stability

Thus, the obtained results demonstrate the potential of neuro-agent systems in biologically inspired modeling tasks and provide a foundation for further experiments aimed at exploring the limits of cognitive complexity under constrained perceptual and control resources.

## Conflict of interest

The authors declare no conflict of interest.
